# The use of intra-operative ultrasound in gynecological surgery: a review

**DOI:** 10.2144/fsoa-2020-0172

**Published:** 2021-01-12

**Authors:** Karen Grewal, Benjamin Jones, Ariadne L'Heveder, Sita Jindal, Nicolas Galazis, Srdjan Saso, Joseph Yazbek

**Affiliations:** 1 Tommy's National Centre for Miscarriage Research, Institute of Reproductive & Developmental Biology, Imperial College London, Hammersmith Hospital Campus, Du Cane Road, London, UK; 2 Department of Metabolism, Digestion & Reproduction, Institute of Reproductive & Developmental Biology, Imperial College London Hammersmith Hospital Campus, Du Cane Road, London W12 0NN, UK; 3 Queen Charlotte's Hospital, Hammersmith Hospital Campus, Imperial College London, Du Cane Road, London W12 0HS, UK

**Keywords:** benign, gynecological oncology and fertility sparing surgery, gynecology, intraoperative ultrasound

## Abstract

Ultrasound is a readily available, safe and portable imaging modality that is widely applied in gynecology. However, there is limited guidance for its use intra-operatively especially with complex gynecological procedures. This narrative review examines the existing literature published on the use of intraoperative ultrasound (IOUS) in benign gynecology and in gynecological oncology. We searched for the following terms: ‘intraoperative,’ ‘ultrasonography,’ ‘gynecology’ and ‘oncology’ using Pubmed/Medline. IOUS can minimize complications and facilitate difficult benign gynecological procedures. There is also a role for its use in gynecological oncology surgery and fertility-sparing surgery. The use of IOUS in gynecological surgery is an emerging field which improves visualization in the surgical field and aids completion of minimally invasive techniques.

## Lay abstract

Ultrasound (US) is a portable and safe imaging method that uses high frequency sound waves to visualize structures within the body. While most US examinations are done outside the body there is an emerging field which uses US devices within the body during surgery to aid complex procedures. This review examines the published literature on this technique in benign gynecology and in gynecological oncology. This review demonstrates the use of intraoperative US improves visualization and minimizes surgical complications.

Ultrasound (US) is used extensively within the field of obstetrics and gynecology (O&G). It can facilitate the diagnosis and monitoring of various gynecological conditions. In addition, US has revolutionized obstetric care in the detection of structural abnormalities, and in the management of small for gestational age fetuses [1]. US is widely available throughout early pregnancy and gynecology clinics and is essential in the diagnosis and management of early pregnancy disorders, benign and malignant gynecological problems. In the UK, US has become an integral component of the O&G trainee curriculum. The implementation of competency-based assessments ensures that the necessary expertise is attained, reinforcing its centrality to the field.

Intraoperative US (IOUS) has become increasingly common across a wide range of specialties since its introduction in 1961 to facilitate the detection of renal calculi [2]. It has since been used within the field of gynecology to facilitate various operations from procedures as minor as removing a lost contraceptive implant [3–5] or intrauterine device [6], to more complex, invasive procedures including surgical termination of pregnancy, hysteroscopy and laparoscopic myomectomy [7–9]. More recent developments in US technology have resulted in greatly enhanced image quality and real-time IOUS has become increasingly utilized within the gynecological setting.

Minimally invasive surgical techniques, such as laparoscopy, are becoming more prevalent because of improved operator skill and the continued reported benefits to the patient. However, the lack of direct tissue palpation leads to challenges related to intra-operative tactile feedback [10]. Furthermore, while pre-operative imaging can allow extensive mapping and procedural planning, it does not compare to the potential information that real-time imaging can provide. This is exemplified by Levine *et al.* who showed that intra-operative laparoscopic US probes were able to demonstrate more myomas during myomectomy than pre-operative trans-vaginal US (TVS) or magnetic resonance imaging (MRI) scan [11]. This reinforces the need for IOUS as an adjunctive operating tool to provide additional information to the surgeons. This could theoretically improve surgical accuracy, reduce complications, and ultimately improve patient care.

This review examines the existing literature published on the use of IOUS in gynecological surgery. We searched for the following terms, ‘intraoperative,’ ‘ultrasonography,’ ‘gynecology’ and ‘oncology’ using Pubmed/Medline. We only included studies written in English that illustrated the clear use of IOUS in gynecological surgery.

## Benign gynecology

### Surgical termination of pregnancy & management of miscarriage under US guidance

The surgical management of termination of pregnancy and miscarriage varies, not only because of operator choice of instruments and techniques, but also the gestation of the pregnancy and the setting it is being carried out.

Dilation and curettage (D&C), with or without suction/vacuum aspiration, is a frequently performed gynecological procedure, making its safety paramount [7,10]. However, it is historically carried out blindly with an early complication rate of approximately 6% [12]. Such early complications include uterine perforation, excessive bleeding greater than 500 ml, retained products of conception necessitating further intervention and pelvic infection [12–14]. The benefits IOUS can provide when performing this procedure are well established. The use of continuous concurrent IOUS significantly reduces the complication rate in both the operating room and in the office-based setting [10,15–17]. A randomized control led trial of 248 women conducted by Acharya *et al.* showed significantly reduced complication rates during surgical termination of pregnancy under US guidance when compared with those carried out without US guidance (3.7 vs 15.9%; RR: 0.23; CI: 0.08–0.67). The group with US guidance demonstrated significantly reduced rates of infection, retained products of conception requiring repeat evacuation procedure, intra- and post-operative blood loss, procedure duration and convalescence time. The study concluded that eight patients required US guidance to prevent one additional complication (95% CI: 5–23) [7].

Complications with surgical termination of pregnancy and surgical management of miscarriage procedures carried out in the second trimester are similar to those in the first trimester but often lead to more serious consequences. Bleeding increases with gestational age and uterine perforation may require an exploratory laparotomy as cases can be associated with bowel injury or hemorrhage due to the larger size of instruments used [18]. Current evidence suggests there are additional advantages associated with the use of IOUS in the second trimester as it assists with complete amniotic fluid drainage and locating various fetal parts for removal [19,20].

### The diagnosis & management of molar pregnancy

Gestational trophoblastic disease is a group of interrelated tumors that arise from the placental villous trophoblast. The term hydatidiform mole refers to abnormal trophoblastic proliferation and can be characterized as complete (diploid in karyotype without fetal tissue and only derived from the paternal genome) or partial (triploid in karyotype from both maternal and paternal genomes) [21]. The early diagnosis and recognition of molar pregnancy is important to plan surgical management and pre-empt intraoperative complications such as hemorrhage. The early pregnancy detection rates for TVS in molar pregnancy range from 50 to 86%. The limited use of TVS is often due to the fact morphological features of hydropic villi are only confirmed on histological examination and hence this is still the gold standard for diagnosis. The main features on US represent a classical ‘snowstorm’ appearance with multiple anechoic and hypoechoic spaces. There are limited reports in the literature of the use of US guidance in the surgical evacuation of molar pregnancy. The use of repeated evacuation for persistent disease is often not recommended and in such cases chemotherapy is indicated. Nonetheless some case reports suggest IOUS is useful in cases of myometrial invasion and ensuring complete evacuation [22,23].

### Management of cesarean scar pregnancy with US

There is limited consensus on the optimum management of cesarean scar pregnancies (CSP). The main management options include: expectant, medical (with local or systemic methotrexate) and surgical (dilatation and suction curettage with additional hemostatic measures such as the Cook balloon, Shirodkar suture and uterine artery embolization) [24–29]. Comparability of management techniques within studies is difficult given the lack of clear diagnostic test criteria. There is a drive to upload cases to an International CSP Registry to standardize management and diagnostic protocols [30]. A recent national cohort study in the UK which included the management of 102 CSPs found that surgical management with dilation and suction curettage (under US guidance in the majority of cases) was associated with the highest success rates and lowest complications. The success rate of medical and expectant management was 47 and 43%, respectively [24]. A recent systematic review of CSP treatment options also concluded that dilation and suction curettage in selected groups was favored over medical treatment [31]. Studies that use dilation and curettage with US guidance have shown success rates of 79.7% and favor the use of US over hysteroscopy and concomitant laparoscopy. US is considered essential for the safety and success of the procedure as it minimizes procedure time and reduces the risk of retained tissue and perforation [28,29]. Most studies use a transabdominal approach although transrectal US (TRUS) has also been suggested to visualize the entire uterine cavity. The use of TRUS allows more freedom of movement with the suction cannula compared with the restrictions of TVS [32]. Local methotrexate (MTX) injection under TVS guidance has also been shown to be a safe, successful and effective in treatment of CSP with appropriate case selection. Although the prolonged treatment course and risk of recurrence needs to be considered when discussing the treatment options with the patient [33,34]. A recent retrospective cohort study assessed the factors associated with treatment success in cases using aspiration and local MTX in CSP. They concluded that early detection of CSP with low beta HCG levels are associated with successful outcomes when using this method [35]. Nonetheless there is insufficient evidence to conclude whether a particular management strategy can impact reproductive outcome after CSP [36]. Therefore, ongoing discussion and research is required to evaluate the optimum treatment strategy and the use of IOUS should be formally evaluated in this field.

### US guided hysteroscopic procedures

Hysteroscopy is extensively applied in both the diagnosis and treatment of many gynecological conditions such as resection of fibroids, polyps, removal of uterine adhesions and metroplasty [10]. One of the main challenges associated with operative hysteroscopy is poor visualization. This is especially the case with Asherman's syndrome where scarring within the cavity impedes visualization and increases the risk of uterine perforation. Cervical stenosis is another encountered problem in hysteroscopy that is carried out to investigate postmenopausal bleeding. The failure to gain access into the uterine cavity in those patients may lead to unnecessary recommendation for hysterectomy in view of the possibility of endometrial cancer [37]. In our experience IOUS via transabdominal or transrectal route is effective in guiding the operating surgeon into the uterine cavity. The use of concurrent real-time US guidance can facilitate operative hysteroscopy with minimal complications and is considered more efficient and favorable than the use of adjunctive laparoscopy [8]. A prospective study of 120 women with a single submucosal fibroid undergoing hysteroscopic resection with and without TRUS was conducted to evaluate complete resection at 4–8 weeks postoperatively. The use of TRUS with operative hysteroscopy was associated with complete resection (area under curve 0.8; p < 0.001) [38]. In such cases IOUS can visualize the deepest margin of the myoma and obtain complete excision while protecting the integrity of the uterus and preventing perforation [38,39].

### US guidance in laparoscopic procedures

Laparoscopy is the gold standard for many gynecological procedures and the use of US can be a beneficial operative tool. A recent meta-analysis showed that laparoscopic myomectomy is associated with lower post-operative fever, pain and shorter hospital stay than open procedures [40]. However, other studies have reported a higher incidence of fibroid recurrence which have been attributed to the lack of palpation which can make it difficult to locate and extract all leiomyomas during laparoscopy [41,42]. In light of this, IOUS has great potential in assisting myomectomies conducted laparoscopically. In 2004, Lin *et al.* first described the use of an intra-operative laparoscopic US probe (LUS), in order to locate and resect an intramural fibroid. This fibroid was not visible laparoscopically because of the normal outer contour of the uterus. The use of US allowed a successful myomectomy to be performed, preventing the need to convert to open surgery [9]. These benefits have been further investigated and reproduced in a number of prospective studies which confirm the ability of IOUS (endoscopic or transvaginal) to accurately localize and remove additional myomas that are not visible laparoscopically [43,44]. A recent study investigating the use of IOUS laparoscopically and transvaginally found LUS was more effective in localizing residual fibroids following laparoscopic myomectomy [45].

The use of IOUS in fertility sparing surgery (FSS) is also a valuable tool. Anti-*N*-methyl-d-aspartate receptor (NMDAr) encephalitis is a potentially fatal condition associated with ovarian teratomas in 50% of cases [46]. This condition requires timely diagnosis and removal of the precipitating teratoma [46,47]. Laparoscopy can be complicated as small cysts are not always visible laparoscopically, making FSS in young women of reproductive age challenging. One published case report illustrates the use of transvaginal IOUS to facilitate the removal of an ovarian teratoma in a 29-year-old woman with anti-NMDAr encephalitis, in whom the lesion was not visible laparoscopically [48]. The use of IOUS enabled full excision of the lesion, without cyst rupture and maximal preservation of normal ovarian cortex. A suggested algorithm for the diagnosis and management of such cases was subsequently proposed, recommending the use of IOUS for the management of women with anti-NMDAr encephalitis associated with ovarian teratomas, which are too small to be visualized by laparoscopy alone [48]. A recent systematic review exploring IOUS in FSS demonstrated that such cases benefited from improved complete resection and complication rates as well as subsequent pregnancy. Although larger studies are required to validate these outcomes the findings of the review are overall promising and encourage the application of IOUS in FSS [49].

## Gynecological oncology surgery

The use of IOUS in gynecological–oncological procedures is far less established than its use in benign gynecology. The use of IOUS has however been reported in the detection of metastases, identification of cancers too small to be visualized laparoscopically, assessment of tumor invasion into pelvic organs and the use of IOUS for guidance of intracavitary brachytherapy for cervical cancer.

### Liver metastases

There is mounting evidence that IOUS may facilitate the identification and treatment of liver metastases. However, most of the studies evaluating this potential role are in the context of colorectal cancer rather than gynecological cancers [50]. One study from 1994 did, however, include patients with ovarian cancer (8 out of 110 patients) and found overall that IOUS detected 37 lesions not seen on CT in 21 patients (19%) and 13 lesions not detected by bimanual palpation in 6 patients (5%) [51]. A review on intra-operative imaging concluded that IOUS detects more liver metastases than pre-operative US or CT [50]. Indeed, *Meijer* *et al.* found that IOUS in combination with palpation of the liver was significantly more accurate than pre-operative computed tomography (CT) or percutaneous US [52]. A more recent study reaffirmed the role of IOUS in this setting by demonstrating that despite modern cross-sectional pre-operative imaging, IOUS detected additional liver metastases in 10% of the study patients (n = 213). The authors felt that detection of additional disease, not identified pre-operatively, may provide additional insight to alter the surgical approach which may facilitate complete removal of disease and therefore may improve outcomes. While these studies were carried out in patients with colorectal cancer, it is logical that the findings could be extrapolated for the assessment of liver metastases intraoperatively for gynecological malignancies, although this requires further investigation [53].

### Serous borderline ovarian tumors excision

Jones *et al.* explored the use of IOUS in the removal of recurrent serous borderline ovarian tumors that are too small to be visualized laparoscopically [54,55]. The group proposed the use of IOUS to facilitate laparoscopic ovarian wedge resection which minimizes the amount of healthy ovarian tissue being excised, reducing the likelihood of premature ovarian insufficiency post-operatively. The increased accuracy associated with the use of IOUS reduces the risk of intra-operative cyst rupture, which is associated with increased recurrence risk [55]. The use of continuous intraoperative trans-vaginal US (TVS) was piloted in one patient who was disease free at 18 months with an anti-Mullerian hormone concentration of 9.3 pmol/l [54]. A further study including seven women, six of whom remained disease-free and had resumed normal menstrual function with no clinical or biochemical evidence of premature ovarian insufficiency [55]. Since publication three pregnancies have been documented in those patients: one resulted in first trimester miscarriage, one set of twins and one live birth. This approach offers a fertility-preserving option for women who may otherwise have had an oophorectomy.

### Assessment of myometrial invasion

A further potential use for IOUS is in the assessment of myometrial invasion (MI) in endometrial cancer. There is ongoing debate regarding the value of lymph node dissection in low-risk endometrial cancers. It is currently recommended but the evidence suggests it may not improve survival and the associated morbidity of lymph node dissection in low-grade endometrial cancers may outweigh the benefit [56,57].

Measuring the depth of MI may help determine the necessity of concomitant lymph node dissection. Basaran *et al.* recently carried out a prospective study assessing the diagnostic accuracy of a novel intraoperative *ex vivo* high-resolution sonography (IEHVS) technique in determining the depth of MI in patients with low-risk endometrial cancer. They found that IEVHS correctly assessed the depth of MI in 39 of 45 cases (86.6%), overestimated it in five cases (11.1%), and only underestimated one case of deep MI. These results of IEVHs were compared with frozen sections (FS) finding overall similar accuracies for both techniques. IEVHS did however show a higher (non statistically significant) sensitivity for detecting deep MI compared with FS (87.5 vs 62.5%). The study concludes that IEHVS could be a useful adjunct to FS with improved accuracy by guiding which part of the myometrium to target for FS [58].

### Management of small placental site trophoblastic tumor

Placental site trophoblastic tumors (PSTT) are rare variations of gestational trophoblastic disease that are more aggressive in nature and slow growing. They are often resistant to chemotherapy and hence the primary treatment is often surgical and involves a total abdominal hysterectomy with pelvic and retroperitoneal lymph node sampling [59,60]. Saso *et al.* used contact US during open surgery for the management of a presumed single nodule of PSTT in a patient wishing to preserve her fertility. Intra-operative TVS allowed accurate hysterotomy and minimized excision of healthy tissue. This technique prevented radical surgery in a young patient wishing to preserve her fertility. The patient subsequently conceived naturally and delivered at term by cesarean section [61].

### Intraoperative sonographic guidance for intracavitary brachytherapy of cervical cancer

The most established use for IOUS in the gynecological oncological setting appears to be in the selection and application of intracavitary brachytherapy via an applicator for cervical cancer. Correct applicator placement is crucial in achieving local tumor control [62]. A recent retrospective review assessed the role of IOUS in 142 patients who received tandem-based intracavitary brachytherapy for cervical cancer over 5 years. The study found that with sonographic guidance only one out of 113 patients had uterine perforation (0.9%), compared with two out of 29 (6.9%) when US was not used [63]. This study reiterated previous work that demonstrated the successful use of IOUS in applicator placement [64,65]. Nonetheless, despite the obvious benefits, the use of IOUS in guiding tandem placement is not routinely practiced [65]. The reason for this is unclear, particularly as the use of IOUS shortens the duration of tandem-brachytherapy placement as well as minimizes complications [66].

### Laparoscopic US in gynecological oncology

There are limited data on the use of laparoscopic US (LUS) in gynecological oncology. A previous review suggested LUS provides improved visualization of anatomy and reduces complication and re-intervention rates, but its use has been limited to benign gynecology [10]. Yang *et al.* investigated the ability of LUS to characterise adnexal masses compared with pre-operative TVS in 58 women undergoing laparoscopies for suspected adnexal masses. They demonstrated the accuracy of LUS for characterising adnexal masses was significantly higher than that of pre-operative TVS (83.8 vs 73.5%, p < 0.05). However, the study did not identify improvement in diagnostic accuracy for borderline or malignant lesions as there were too few of these cases to achieve statistical power [67]. Gong *et al.* found LUS to be very beneficial in lesion segmentation in benign gynecology cases, highlighting the major benefit to be image representation beyond tissues boundaries [68]. However, while the 42 women in the study were benign, the obvious potential to extrapolate into the gynecological oncology setting is clear, although further evidence is needed.

## Conclusion

**Figure 1.**
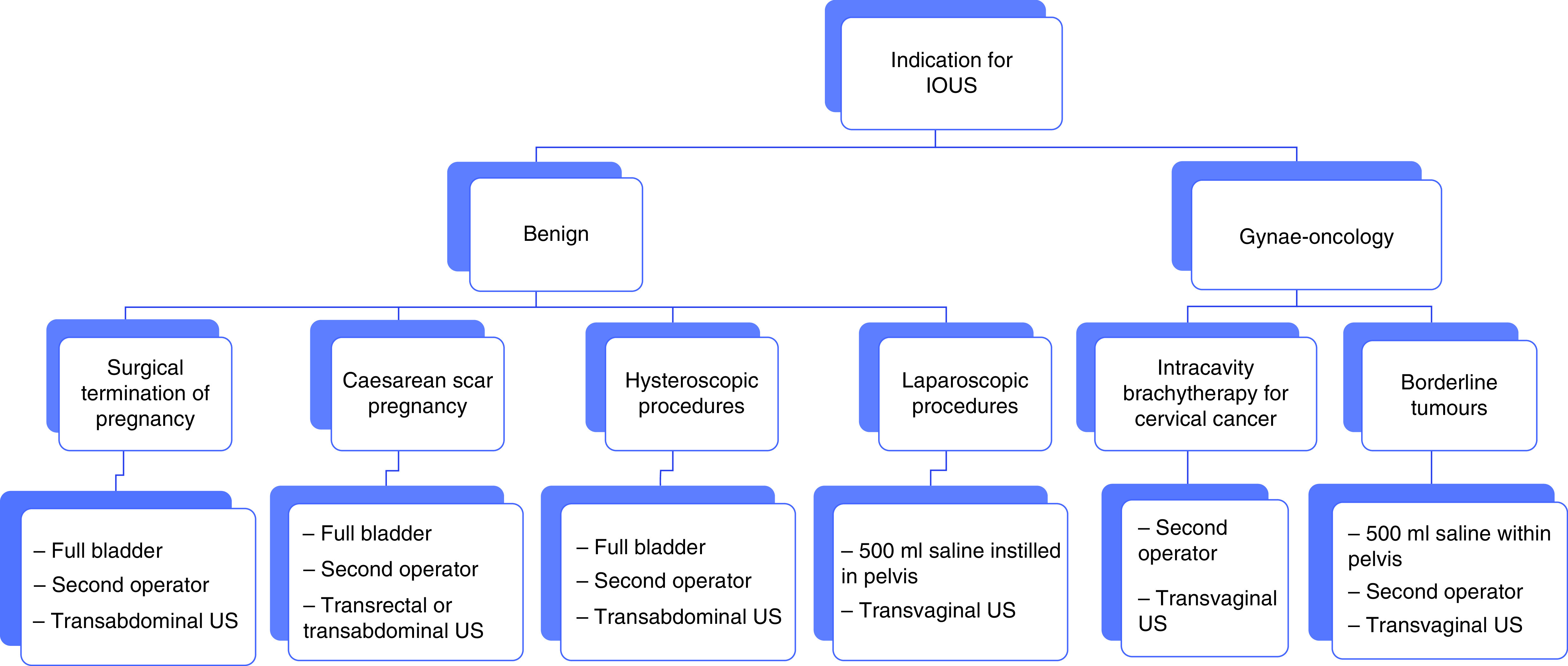
Demonstrates the variety of indications for the use of intraoperative ultrasound in benign and gynecological oncology settings as well as outlining additional considerations. IOUS: Intraoperative ultrasound; US: Ultrasound.

As summarized herein, the main advantage of incorporating IOUS into gynecological surgery is to improve visualization, thus facilitating the completion of procedures through less invasive routes. Figure 1 demonstrates the gynecological benign and oncological indications for the use of IOUS as well as the additional considerations that are required for each case. IOUS is portable, does not impose any additional risk to the patient and has a low economic burden [65]. Although it is unclear whether additional information regarding pathology is identified with IOUS, the benefits in assisting the surgeon to prevent complications and to facilitate accurate incision placement in endoscopic procedures is well documented [69]. While promoting its potential benefits one must however be mindful that, as an imaging modality, US is subject to a high degree of operator dependency, with significant expertise required in order to obtain and interpret images correctly. Furthermore, IOUS would require a degree of US skill that should be possessed by the operating surgeon. The use of TAUS may also require an additional health care professional and views may be limited by BMI and abdominal scarring. Therefore, the developments of LUS, TVS assisted device and TRUS alongside improved equipment and imaging modalities will certainly widen the scope of its use.

## Future perspective

IOUS is a new and evolving field with a wide range of advantages that facilitate complex gynecological procedures. The future of this discipline involves expanding its use and integrating this tool into surgical training to ensure appropriate skills are developed by the operating team. Future research involves creating a machine learning diagnostic US tool to aid the surgeon in the intraoperative setting. Finally, telemedicine will allow acquisition of expert opinion in real time without requiring the expert to be present.

**Executive summary**

**Background**

- Intraoperative ultrasound (IOUS) has been used within gynecology to facilitate a wide range of operations.

**Surgical termination of pregnancy and management of miscarriage under ultrasound guidance**

- Randomized control trials have shown reduced complication rates during surgical termination of pregnancy under ultrasound (US) guidance compared with those that were carried out without US guidance.

**The diagnosis and management of molar pregnancy**

- The early pregnancy detection rates for molar pregnancy using transvaginal US are limited as the specific morphological features are only confirmed on histological examination.
- Some reports show IOUS can be useful to ensure complete evacuation during surgical management of molar pregnancy to minimize retained tissue.

**Management of cesarean scar pregnancy with US**

- Recent studies have shown surgical management with dilation and suction curettage (under US guidance in the majority of cases) are associated with the highest success rates and lowest complications.

**US-guided hysteroscopic procedures**

- IOUS can facilitate operative hysteroscopy by enhancing visualization and allow complete resection of submucosal fibroids.

**US guidance in laparoscopic procedures**

- Intraoperative laparoscopic US probes can help locate and resect intramural fibroids that would otherwise not have been visible laparoscopically.

**Gynecological oncology surgery**

- The use of IOUS is less established in gynecological oncological procedures.

**Liver metastases**

- Studies mainly focused on colorectal cancer have shown that IOUS detected additional liver metastases compared with pre-operative imaging.

**Serous borderline ovarian tumors excision**

- IOUS can facilitate laparoscopic ovarian wedge resection which minimizes the amount of healthy ovarian tissue being excised and reduces the risk of premature ovarian insufficiency in women wishing to preserve their fertility.

**Assessment of myometrial invasion**

- IOUS can assess myometrial invasion in endometrial cancer and determine if lymph node dissection is required.

**Management of small placental site trophoblastic tumor**

- Placental site trophoblastic tumors are rare variations of gestational trophoblastic disease.
- IOUS can facilitate fertility sparing surgery in patient with a single nodule of placental site trophoblastic tumor.

**Intraoperative sonographic guidance for intracavitary brachytherapy of cervical cancer**

- The use of IOUS has been shown to minimize perforation and ensure adequate placement for intracavitary applicators for brachytherapy in cervical cancer.

**Laparoscopic US in gynecological oncology**

- There are limited data on the use of laparoscopic US in gynecological oncology.
- Studies have shown laparoscopic US improves visualization of anatomy and reduces complication and re-intervention rates.

**Conclusion**

- IOUS is portable and safe, but is subject to operator dependency, with significant expertise required to interpret images correctly.
- IOUS facilitates completion of procedures through less invasive routes.
